# Naps, night-time sleep and cognitive function among middle-aged and older people in China

**DOI:** 10.1371/journal.pone.0328367

**Published:** 2025-07-23

**Authors:** Xiuxiu Zhou, Yutang Tan, Di He, Hong Wu

**Affiliations:** 1 Wuhan Mental Health Center Affiliated to Tongji Medical College of Huazhong University of Science and Technology, Wuhan, Hubei, China; 2 Department of Social Medicine and Health Management, School of Public Health, Tongji Medical College, Huazhong University of Science and Technology, Wuhan, Hubei, China; 3 Department of neurosurgery, Zhongnan Hospital of Wuhan University, Wuhan, China; 4 School of Medicine and Health Management, Tongji Medical College, Huazhong University of Science and Technology, Wuhan, China; Sapienza University of Rome: Universita degli Studi di Roma La Sapienza, ITALY

## Abstract

**Background:**

There is increasing interest in how sleep affects cognitive function; however, the combined impact of naps and night-time sleep on different cognitive domains is still not well understood. This study investigates the relationship between naps, night-time sleep, and cognitive function over time among middle-aged and older adults in China, as well as how this relationship may differ between rural and urban residents.

**Methods:**

A total of 2,938 community residents aged 45 and older were selected from the China Health and Retirement Longitudinal Study (CHARLS, conducted in 2013, 2015, and 2018). The study examined the relationship between napping, night-time sleep, and cognitive function using fixed-effects analysis over a period of five years.

**Results:**

Sleeping 6–8 hours/ night and napping for less than 30 minutes/ day were associated with better cognitive function (*β* = 0.383, 95% CI: 0.198, 0.567) and memory (*β* = 0.304, 95% CI: 0.155, 0.451) across the entire sample. In contrast, sleeping more than 8 hours/ night and napping more than 90 minutes/ day were associated with poor mental status. Specifically, sleeping 6–8 hours/ night was significantly associated with better cognitive function (*β* = 0.501, 95% CI: 0.252, 0.750) and memory (*β* = 0.372, 95% CI: 0.173, 0.572) in rural respondents. Sleeping more than 8 hours/ night was associated with poorer mental status among urban respondents (*β* = −0.291, 95% CI: −0.551, −0.032). Rural respondents who napped less than 90 minutes/ day had improved cognitive function. Napping for more than 90 minutes/ day was significantly correlated with cognitive function and mental status, which was primarily observed among urban respondents.

**Conclusions:**

Considerable differences were observed between rural and urban areas regarding the relationship between napping, night-time sleep, and cognitive function. When designing interventions to enhance cognitive function, it’s essential to take into account cultural context, geographical factors, and individual differences.

## Introduction

As the global population ages, age-related dementia has emerged as a significant public health issue [1]. Currently, dementia affects 6–50 million people worldwide, and its prevalence appears to double every 5 years for individuals aged 50–80 [2]. In China, approximately 5% to 30% of older adults are affected by dementia [3]. This condition substantially increases the risk of functional dependence and decrease the quality of life for middle-aged and older adults. It also places a heavy burden on families and presents considerable challenges to healthcare systems and society as a whole [4]. Given that cognitive decline is considered a precursor to dementia [5], it is essential to understand the factors contributing to cognitive decline in order to improve cognitive function and prevent further deterioration.

Changes in sleep patterns are common among older adults, and the prevalence of sleep disorders linked to disrupted sleep and fragmentation tends to increase with age [6]. These age-related alterations in sleep could contribute to cognitive decline, although this connection has not been thoroughly investigated. Previous research has indicated that adequate sleep duration is crucial for optimal cognitive function [7]. There may be a mechanism that explains how sleep affects cognitive performance, as sleep plays a role in regulating the inflammatory response [8]. Normal nighttime sleep is associated with reduced sympathetic nervous system activity, while short sleep durations may activate the sympathetic pathway, leading to increased inflammation. This inflammation can be reflected in markers such as white blood cell counts and C-reactive protein (CRP), which may affect brain structure, including cortical changes and reductions in gray matter volume, potentially contributing to cognitive impairment [9]. Additionally, the Synaptic Homeostasis hypothesis suggests that sleep helps to normalize synaptic strength and maintain brain homeostasis [10]. During wakefulness, synapses are strengthened through learning, and sleep helps to prune unnecessary connections, thereby facilitating the processing and retention of important information. The Glymphatic System hypothesis posits that sleep aids in clearing metabolic waste from the brain, including harmful proteins like β-amyloid, which are linked to neurodegenerative diseases [11]. Moreover, the Attention Restoration theory argues that sleep replenishes cognitive resources and enhances attention control, both of which are essential for performing tasks that require sustained concentration [12]. However, research has also identified an inverted U-shaped relationship between sleep and overall cognitive decline, suggesting that both insufficient and excessive sleep duration can negatively impact cognitive function [13]. Therefore, maintaining healthy sleep patterns is vital for enhancing cognitive function in middle-aged and older adults.

Rapid economic development, urbanization, and sociodemographic and lifestyle factors have a significant impact on sleep patterns [6] and cognitive function [14]. The swift and disproportionate economic growth in both urban and rural areas of China has led to unexpected public health challenges. While numerous studies have explored the overall relationship between sleep and cognitive function [13,15], regional differences still persist. Evidence suggests that low socioeconomic status and poor living conditions in rural areas are associated with increased sleep fragmentation [16], potentially contributing to cognitive decline [17]. Furthermore, cultural differences can influence cognitive performance, leading to variations between rural and urban populations [6]. Despite previous research identifying a connection between sleep and cognitive function, the findings in the literature are inconsistent. For instance, a survey of 2,751 community-dwelling older men over 12 years revealed that those who napped longer experienced greater cognitive decline and a higher risk of cognitive impairment [18]. A study involving 3,132 community-dwelling older men from six centers in the United States found that those who slept for more than eight hours had a 0.6-point lower score on the Mini-Mental State Examination [19]. However, research from the UK Biobank, which included 479,420 participants aged 38–73, indicated that seven hours of sleep per night was associated with the highest cognitive performance [20]. Moreover, previous studies have shown that patients with insomnia disorders tend to have delays in performing visual tasks [21] and exhibit significant changes in executive functioning and episodic memory [22]. While much of the focus has been on nighttime sleep, the effects of afternoon napping on cognition have not been thoroughly examined. The combined influence of napping and nighttime sleep on various cognitive domains remains unclear.

Thus, this study aimed to explore the relationship between naps, night-time sleep, and two aspects of cognitive function: memory and mental status. Additionally, given the significant socioeconomic disparities between rural and urban areas, as well as the notable urban-rural differences in China that impact naps, night-time sleep, and different dimensions of cognitive function [23,24], we further examined these associations within urban and rural subgroups to gain a deeper understanding of the Chinese context.

## Methods

### Study sample

The data used in this study were obtained from the China Health and Retirement Longitudinal Study (CHARLS), conducted from 2013 to 2018. CHARLS is a nationally representative longitudinal survey focused on middle-aged and older individuals. It is carried out by the China Centre for Economic Research of Peking University using a probability-proportional-to-size sampling technique. This approach covers 450 villages and 150 counties across 28 provinces, and data are collected through face-to-face interviews after participants have given their informed consent [25]. The CHARLS has received ethical approval from the Institutional Review Board of Peking University (ethical approval number IRB 00001052–11015). In CHARLS 2013, a total of 18,448 community-dwelling residents participated. We first excluded 14,251 respondents who were younger than 45 years. Next, we removed 784 respondents who were lost to follow-up in CHARLS 2015, as well as 475 respondents lost to follow-up in CHARLS 2018. As a result, 2,938 respondents remained for the fixed-effects regression analysis, and we applied multiple imputation to address the missing data.

### Variables

#### Cognitive function.

The cognitive function items in CHARLS were derived from the Telephone Interview of Cognitive Status (TICS) battery, which is similar to the one used in the US Health and Retirement Study [26]. The TICS serves as a telephone version of the Mini-Mental State Examination and has been validated for assessing cognitive function [27,28]. Previous studies have suggested that cognitive function can be measured along two dimensions: memory and mental status [24,29,30]. CHARLS evaluated memory using two tasks. First, respondents were asked to immediately recall and repeat 10 Chinese nouns that were just read to them, with responses allowed in any order. This immediate recall was followed by a delayed recall task, where respondents had to repeat the same words four minutes later. Scores for the memory component ranged from 0 to 20 points. CHARLS assessed mental status through three subcomponents: time orientation, visuoconstruction, and numeric ability. Time orientation, with a maximum of 5 points, was evaluated by asking respondents to identify the current date (year, month, and day), the season, and the day of the week. Visuoconstruction was measured by asking respondents to accurately redraw a previously shown picture, which could earn them up to 1 point. Numeric ability was assessed by having respondents subtract 7 from 100 consecutively for five rounds, with a maximum score of 5 points. The total cognitive function score, derived from the sum of memory and mental status scores, ranges from 0 to 31, with higher scores indicating better cognitive function.

#### Sleep.

Two sleep-related variables were examined: afternoon napping and night-time sleep.

**Afternoon Napping:** In CHARLS, participants were asked, “During the past month, how long did you take a nap after lunch?” Based on findings from previous studies [31], napping duration was categorized into four groups: no napping, less than 30 minutes, 30–90 minutes, and more than 90 minutes.

**Night-time Sleep:** This variable was measured by asking the following question: “During the past month, how many hours of actual sleep did you get at night (average hours for one night)? (This may be shorter than the number of hours you spend in bed.)” According to the existing literature [32], night-time sleep duration was classified into three groups: less than 6 hours, 6–8 hours, and more than 8 hours.

#### Control variables.

The control variables were categorized into three main aspects: socio-demographic characteristics, health behaviors, and health status-related variables. For socio-demographic characteristics, we included gender (male, female), age, residency (rural, urban), education (illiterate, primary school and lower, middle school, senior middle school and higher), marital status (single, partnered), retirement (no, yes) and living near children (no, yes). For health behavior-related variables, we included smoking (no, yes) and alcohol consumption (no, yes). Health status-related variables included hypertension (no, yes), diabetes (no, yes), cancer (no, yes), hearing impairment (no, yes), stroke (no, yes), depression (no, yes), and instrumental activities of daily living (IADLs). Depression was assessed using the 10-item Centre for Epidemiologic Studies Depression Scale (CES-D Scale), with a cut-off score of 10 indicating a risk of depression [33]. An IADL impairment was defined as having difficulty with one or more of the five IADL items.

### Data analysis

The characteristics of the overall sample and the rural/urban groups were described using the mean value and standard deviation (SD) for continuous data, while the percentages were used for categorical data. We examined differences between rural and urban respondents using a t-test for continuous variables and a chi-square test for categorical variables. After adjusting for potentially confounding variables, we used a longitudinal linear fixed-effects regression model to assess the relationship between naps, night-time sleep and cognitive function in both urban and rural participants. This model treats each individual as their own control, allowing us to investigate how changes in the independent variable affect the dependent variable.


$${Cognitive\;function}_{it}=\mu_{t}+\beta_{1}{sleep}_{it}+\beta_{2}X_{it}+\alpha_{i}+\varepsilon_{it}$$

*Cognitive function*_*it*_ indicates the cognitive performance of individual *i* at time *t*. *Sleep*_*it*_ suggests the afternoon napping and total night-time sleep duration for individual *i* at time *t*. *X*_*it*_ denotes time-varying variables, such as health behaviors and health outcome variables. *μ*_*t*_ captures year-specific effects, whereas *α*_*i*_ characterizes all time-invariant variables.

The F-test and Hausman test were employed to select the appropriate model among the random-effects model, ordinary least squares (OLS), and fixed-effects model. The F-test and Hausman test yielded statistically significant results (*p* < 0.001), suggesting that the fixed-effects model was the most suitable choice (R^2^ = 0.045, AIC = 59768.26, BIC = 90435.57). The results are presented using *β*-coefficients and 95% confidence intervals (CIs). The data analysis was conducted using R Version 4.3.1.

### Ethics approval

The CHARLS has received ethical approval from the Institutional Review Board of Peking University (ethical approval number IRB 00001052-11015).

## Results

### Basic characteristics of urban and rural respondents

Table 1 illustrates the basic characteristics of the respondents. Among 2,938 respondents, a greater proportion lived in rural areas (60.42%), with primary school and lower education (64.54%), did not smoke (59.28%), partnered (88.80%), living near children (88.34%), and not retired (74.99%). Compared with urban participants, rural participants had a significantly higher risk of depression (34.89%), more difficulty in IADLs (24.94%), and suffered from poorer cognitive function.

**Table 1:** Sample characteristics of urban and rural respondents at baseline.

	Total (n = 2938)	Urban (n = 1163)	Rural (n = 1775)
	n (%)	n (%)	n (%)
Cognitive function Mean (SD)			
total scores	16.75 (4.58)	17.77 (4.30)	16.00 (4.65)
memory	7.57 (3.40)	8.15 (3.46)	7.20 (3.31)
mental status	8.74 (2.26)	9.23 (1.91)	8.38 (2.42)
Gender			
male	1468 (49.97)	588 (50.56)	880 (49.58)
female	1470 (50.03)	575 (49.44)	895 (50.42)
Education level			
illiterate	698 (23.76)	186 (15.99)	512 (28.85)
≤ primary school	1198 (40.78)	409 (35.17)	789 (44.45)
middle school	637 (21.68)	297 (25.54)	340 (19.15)
≥ high school	405 (13.78)	271 (23.30)	134 (7.55)
Marital status			
divorced/widowed/single	329 (11.20)	127 (10.92)	202 (11.38)
married/partnered	2609 (88.80)	1036 (89.08)	1573 (88.62)
Age			
Mean (SD)	57.06 (10.09)	56.44 (10.02)	58.02 (10.13)
Smoking			
no	1740 (59.28)	704 (60.59)	1036 (58.43)
yes	1195 (40.72)	458 (39.41)	737 (41.57)
Alcohol consumption			
no	1532 (52.27)	598 (51.46)	934 (52.80)
yes	1399 (47.73)	564 (48.54)	835 (47.20)
IADLs			
no difficulty	2305 (78.62)	975 (84.05)	1330 (75.06)
some difficulty	627 (21.38)	185 (15.95)	442 (24.94)
Living near children			
no	333 (11.66)	116 (10.23)	217 (12.59)
yes	2524 (88.34)	1018 (89.77)	1506 (87.41)
Retirement			
no	2114 (74.99)	698 (64.51)	1416 (81.52)
yes	705 (25.01)	384 (35.49)	321 (18.48)
Hypertension			
no	1987 (76.63)	720 (73.02)	1267 (78.84)
yes	606 (23.37)	266 (26.98)	340 (21.16)
Stroke			
no	2524 (97.98)	951 (97.74)	1573 (98.13)
yes	52 (2.02)	22 (2.26)	30 (1.87)
Depression			
no	1851 (69.40)	791 (76.13)	1060 (65.11)
yes	816 (30.60)	248 (23.87)	568 (34.89)
Diabetes			
no	2416 (94.12)	879 (90.43)	1537 (96.36)
yes	151 (5.88)	93 (9.57)	58 (3.64)
Cancer			
no	2542 (99.14)	959 (98.87)	1583 (99.31)
yes	22 (0.86)	11 (1.13)	11 (0.69)
Hearing impairment			
no	2375 (87.70)	954 (90.34)	1421 (86.02)
yes	333 (12.29)	102 (9.66)	231 (13.98)

N.B.

SD, standard deviation.

IADLs, instrumental activities of daily living.

### *Urban*-*rural disparity in naps and night-time sleep*

Table 2 shows the differences in afternoon napping and night-time sleep duration between rural and urban participants from 2013 to 2018. Regarding to napping levels, most respondents either did not take naps or napped for 30–90 minutes/ day after lunch across all three waves of data collection. Among rural participants, the highest proportion consisted of non-nappers, accounting for 44.60%, 44.75%, and 39.82% in the years 2013, 2015, and 2018, respectively. In contrast, urban participants exhibited a higher percentage of those napping for 30–90 minutes after lunch, with rates of 41.81% and 38.78% in 2015 and 2018, respectively. Additionally, regarding night-time sleep duration, the majority of both rural and urban residents reported sleeping for 6–8 hours/ night during the same period.

**Table 2:** Afternoon napping and night-time sleep duration between rural and urban participants in 2013–2018.

	2013		2015		2018	
Afternoon Napping	rural	urban	rural	urban	rural	urban
	n (%)	n (%)	n (%)	n (%)	n (%)	n (%)
no napping	883 (44.60)	717 (41.81)	826 (44.75)	526 (39.91)	706 (39.82)	427 (36.72)
< 30 minutes	148 (7.47)	159 (9.27)	106 (5.74)	77 (5.84)	145 (8.18)	112 (9.63)
30–90 minutes	665 (33.59)	654 (38.13)	673 (36.46)	551 (41.81)	664 (37.45)	451 (38.78)
> 90 minutes	284 (14.34)	185 (10.79)	241 (13.06)	164 (12.44)	258 (14.55)	173 (14.88)
**Night-time Sleep**						
< 6 hours	566 (29.42)	505 (29.81)	532 (29.12)	376 (28.7)	616 (34.72)	384 (33.02)
6–8 hours	1199 (62.32)	1077 (63.58)	1113 (60.92)	841 (64.2)	982 (55.36)	692 (59.50)
> 8 hours	159 (8.26%)	112 (6.61)	182 (9.96)	93 (7.1)	176 (9.92)	87 (7.48)

### *Rural*-*urban differences in the relationship between naps, night-time sleep and different dimensions of cognitive function*

The rural-urban differences in the relationship between naps, night-time sleep and total cognitive function, memory, and mental status are presented in Table 3. In the whole sample, sleeping 6–8 hours/ night was associated with better cognitive function (*β* = 0.383, 95% CI: 0.198, 0.567) and memory (*β* = 0.304, 95% CI: 0.155, 0.451). Sleeping more than 8 hours/ night was associated with poor mental status (*β* = −0.227, 95% CI: −0.398, −0.055). For afternoon napping, respondents who napped less than 90 minutes/ day had better cognitive function (<30 minutes: *β* = 0.517, 95% CI: 0.196, 0.837; 30−90 minutes: *β* = 0.373, 95% CI: 0.188, 0.557) and memory (<30 minutes: *β* = 0.356, 95% CI: 0.099, 0.613; 30−90 minutes: *β* = 0.237, 95% CI: 0.089, 0.384) than those who did not. However, napping more than 90 minutes/ day was associated with poor cognitive function (*β* = −0.305, 95% CI: −0.559, −0.051) and mental status (*β* = −0.171, 95% CI: −0.307, −0.035).

**Table 3:** Rural-urban differences in the relationship between naps, night-time sleep and cognitive function.

	Cognitive function	Mental status	Memory
β (95% CI)			
**Model 1: Whole sample**			
Sleep duration			
< 6h	1		
6-8h	0.383*** (0.198, 0.567)	0.079 (−0.019, 0.178)	0.304*** (0.155, 0.451)
> 8h	−0.298 (−0.608, 0.025)	−0.227** (−0.398, −0.055)	−0.070 (−0.327, 0.186)
Afternoon napping			
no napping	1		
< 30 minutes	0.517** (0.196, 0.837)	0.160 (−0.011, 0.331)	0.356** (0.099, 0.613)
30–90 minutes	0.373*** (0.188, 0.557)	0.135** (0.037, 0.234)	0.237** (0.089, 0.384)
> 90 minutes	−0.305* (−0.559, −0.051)	−0.171* (−0.307, −0.035)	−0.133 (−0.337, 0.070)
**Model 2: Rural**			
Sleep duration			
< 6h	1		
6-8h	0.501*** (0.252, 0.750)	0.129 (−0.010, 0.268)	0.372*** (0.173, 0.572)
> 8h	−0.136 (−0.546, 0.273)	−0.174 (−0.403, 0.055)	0.037 (−0.291, 0.367)
Afternoon napping			
no napping	1		
< 30 minutes	0.661** (0.223, 1.099)	0.150 (−0.094, 0.394)	0.511** (0.159, 0.862)
30–90 minutes	0.399** (0.152, 0.646)	0.209** (0.071, 0.348)	0.189 (−0.008, 0.388)
> 90 minutes	−0.160 (−0.495, 0.174)	−0.064 (−0.251, 0.122)	−0.095 (−0.364, 0.173)
**Model 3: Urban**			
Sleep duration			
< 6h	1		
6-8h	0.247 (−0.028, 0.522)	0.019 (−0.118, 0.157)	0.227* (0.006, 0.448)
> 8h	−0.477 (−0.994, 0.039)	−0.291* (−0.551, −0.032)	−0.185 (−0.599, 0.228)
Afternoon napping			
no napping	1		
< 30 minutes	0.323 (−0.146, 0.793)	0.148 (−0.087, 0.384)	0.174 (−0.202, 0.551)
30–90 minutes	0.331* (0.055, 0.607)	0.040 (−0.097, 0.179)	0.290* (0.069, 0.511)
> 90 minutes	−0.496* (−0.886, −0.106)	−0.302** (−0.497, −0.106)	−0.194 (−0.506, 0.118)

N.B.

*All models were adjusted for gender, age, education level, marital status, retirement, smoking, alcohol consumption, living near children, IADLs, hypertension, diabetes, cancer, hearing impairment, stroke.

CI, confidence interval.

*: p < 0.05.

**: p < 0.01.

***: p < 0.001.

The significant urban-rural disparity was observed in the relationship between naps, night-time sleep and cognitive function. Sleeping 6–8 hours/ night was significantly associated with better cognitive function (*β* = 0.501, 95% CI: 0.252, 0.750) and memory (*β* = 0.372, 95% CI: 0.173, 0.572) in rural respondents. Meanwhile, rural respondents who napped less than 90 minutes/ day had improved cognitive function (<30 minutes: *β* = 0.661, 95% CI: 0.223, 1.099; 30−90 minutes: *β* = 0.399, 95% CI: 0.152, 0.646). However, sleeping more than 8 hours/ night was significantly related to worse mental status (*β* = −0.291, 95% CI: −0.551, −0.032) in urban respondents. Furthermore, urban respondents who napped more than 90 minutes/ day had poor cognitive function (*β* = −0.496, 95% CI: −0.886, −0.106) and mental status (*β* = −0.302, 95% CI: −0.497, −0.106).

## Discussion

### *Urban*-*rural disparities in cognitive function*

In this study, we found that respondents in rural areas had significantly poorer cognitive function, which is consistent with prior studies conducted in China [23]. This may be due to the enormous urban-rural division in economic and welfare resources in the country [34]. Better health conditions and more financial resources may reduce urban respondents’ risk of cognitive decline. In addition, regarding the level of education, the rural group presented many illiterates and education was lower than urbans. With such a different starting point, the final results may have been influenced by this factor.

### Naps, night-time sleep in urban and rural communities

Compared to those who slept less than 6 hours/ night and more than 8 hours/ night, a great proportion of respondents slept 6–8 hours/ night, which is similar to the findings from prior studies in other countries [6,15,35]. Furthermore, among rural participants, non-nappers were the majority, while urban participants napped for 30–90 minutes/ day after lunch. This difference likely stems from variations in economic development and work patterns between the two groups [16,36,37]. Urban residents have regular working hours, whereas a large proportion of rural residents are living with lower education levels and are busy with long-term heavy labor, resulting in minimal rest time and thus no napping habit [38]. Moreover, sleep environment factors such as light exposure, noise, and uncomfortable temperature have been shown to affect sleep duration [6]. Individuals living in rural areas may be exposed to a poor sleep environment, potentially disrupting sleep quality.

### Rural-urban differences in relationship between naps, night-time sleep and different dimensions of cognitive function

In general, our study found that naps and night-time sleep were significantly associated with cognitive function, which is in line with previous studies [13,15]. In addition, we found that there were significant differences in the relationship between naps, night-time sleep and cognitive function in urban and rural areas, which emphasizes the role of rural-urban differences in improving cognitive function among middle- and older-aged Chinese.

Our findings indicated that sleeping more than 8 hours/ night was associated with poor mental status. This may be due to an increase in sleep fragmentation, even when the total amount of nighttime sleep exceeds 8 hours. Increased sleep fragmentation has been linked to higher amyloid-β deposition in cognitively healthy older adults, which can eventually lead to nervous system degeneration and a decline in brain functions related to mental processes [39]. Moreover, participants who self-reported long sleep durations may have perceived their sleep as longer than it actually was due to frequent awakenings throughout the night or because they spent a long time in bed without actually sleeping. Reduced sleep quality has been associated with an increased amyloid-β burden in elderly individuals living in the community. This burden may contribute to neurodegeneration by promoting neuroinflammation and disrupting neurogenesis, particularly in the hippocampal region, which is crucial for maintaining mental status [40]. Additionally, owing to previous studies have reported that the typical sleep duration for adults is 7–8 hours per day [41]; sleep duration of more than 8 hours per day could be associated with elevated levels of inflammatory markers, such as interleukin-6 and CRP, with adverse effects on brain structure and increased risk for poor mental status [42,43]. These findings provide new insights to better understand the relationship between sleep duration and mental status.

The study found that respondents who took a nap less than 90 minutes/ day after lunch performed better cognitive function and memory tests compared to those who did not nap or napped more than 90 minutes/ day. Prior studies also found that people who napped for 30–90 minutes had better word recall–which is a sign of good memory–than people who did not nap or who napped for longer than 90 minutes [44]. One possible explanation for this finding is that individuals who did not take a nap after lunch may be genetically short sleepers and slept less at night [45]. While short sleep duration has been associated with reduced cognitive performance at a population level [46], this does not imply that all habitual short-sleepers are cognitively impaired. Indeed, some individuals may adapt to shorter sleep durations without significant cognitive detriments [47]. Another explanation is that the night-time sleep of extended nappers may be highly fragmented and their sleep efficiency may be lower than others, so they may spend more time to compensate for disturbed night-time sleep [48]. The impairment of normal sleep structure may detrimentally affect one’s brain function regarding digit and verbal memory.

Another interesting finding is that a significant urban-rural disparity was observed in the relationship between naps, nighttime sleep, and cognitive function. From a biological perspective, regular nighttime sleep and naps are associated with reduced sympathetic nervous system activity. In contrast, both short and long sleep durations may activate the sympathetic pathway, leading to increased inflammation and potentially contributing to cognitive impairment [9]. Additionally, regarding the MMSE scores and the potential impact of individuals with possible cognitive impairments in the sample. This may have influenced the results, as the literature indicates that cognitive deficits are associated with micro- and macrostructural changes in sleep patterns [49,50]. Rural environments can expose individuals to tremendous noise and uncomfortable temperatures, disrupting sleep quality and potentially affecting cognitive function [51]. Moreover, rural respondents may experience different environmental stressors such as physical labor, limited access to healthcare, and fewer social services, all of which could lead to sleep patterns differently and be associated with poor cognitive function in both memory and mental status [52]. The educational level also plays a crucial role in cognitive function. Higher education levels are generally associated with better cognitive function and buffer the adverse effects of poor sleep quality [38]. Urban respondents tend to have higher levels of education, which could mitigate some of the negative cognitive effects of sleep disturbances. Conversely, the rural group presented many illiterates, and educational attainment was lower than in the urban group. With such a different starting point, the cognitive function may have been influenced by this factor.

### Strengths and limitations

This study examined the differences between rural and urban populations in terms of the relationships among naps, nighttime sleep, and cognitive function in middle- and older-aged Chinese individuals. The findings revealed the rural environment, intensive work, and limited access to healthcare may be all factors explaining the relationship between sleep disorders and cognitive impairment. Additionally, the urban-rural disparity in this relationship highlights important implications for policy-making. Ultimately, this research reinforces the notion that a one-size-fits-all approach is ineffective.

The current study has several limitations. First, the measurements of night-time sleep duration and napping duration were self-reported and we could not obtain data from sleep questionnaires or objective tools like actigraphy to compare the differences in sleep measurements. Second, the data such as frequency, intensity, and quality of night-time sleep and naps, as well as information on habitual short- and long-sleepers, were unavailable, which limited our research. Third, because sleep has immediate effects on cognitive function that may diminish over time, the causal relationship between sleep and cognition requires further investigation. Lastly, while the MMSE is commonly used for cognitive screening, it is a basic and general measure of cognitive function and does not capture the full spectrum of cognitive abilities.

## Conclusions

Based on representative surveys, we found that moderate night-time sleep duration and afternoon napping were generally linked to better cognitive function. However, the strength of this relationship varied depending on the specific duration of sleep and the different aspects of cognitive function being measured. Furthermore, significant differences were observed between urban and rural respondents regarding the effects of naps and nighttime sleep on cognitive function. Moderate nighttime sleep and naps were found to be more beneficial for individuals living in rural areas.

## Abbreviations

CHARLS: China Health and Retirement Longitudinal Survey
CRP: C-reactive protein
TICS: telephone interview of cognitive status
IADLs: instrumental activities of daily living
CES-D: centre for epidemiologic studies depression
SD: standard deviation
